# Impact of a Moving Noise Masker on Speech Perception in Cochlear Implant Users

**DOI:** 10.1371/journal.pone.0126133

**Published:** 2015-05-13

**Authors:** Tobias Weissgerber, Tobias Rader, Uwe Baumann

**Affiliations:** Audiological Acoustics, ENT Department, University Hospital Frankfurt, Frankfurt am Main, Germany; University of Salamanca- Institute for Neuroscience of Castille and Leon and Medical School, SPAIN

## Abstract

**Objectives:**

Previous studies investigating speech perception in noise have typically been conducted with static masker positions. The aim of this study was to investigate the effect of spatial separation of source and masker (spatial release from masking, SRM) in a moving masker setup and to evaluate the impact of adaptive beamforming in comparison with fixed directional microphones in cochlear implant (CI) users.

**Design:**

Speech reception thresholds (SRT) were measured in S_0_N_0_ and in a moving masker setup (S_0_N_move_) in 12 normal hearing participants and 14 CI users (7 subjects bilateral, 7 bimodal with a hearing aid in the contralateral ear). Speech processor settings were a moderately directional microphone, a fixed beamformer, or an adaptive beamformer. The moving noise source was generated by means of wave field synthesis and was smoothly moved in a shape of a half-circle from one ear to the contralateral ear. Noise was presented in either of two conditions: continuous or modulated.

**Results:**

SRTs in the S_0_N_move_ setup were significantly improved compared to the S_0_N_0_ setup for both the normal hearing control group and the bilateral group in continuous noise, and for the control group in modulated noise. There was no effect of subject group. A significant effect of directional sensitivity was found in the S_0_N_move_ setup. In the bilateral group, the adaptive beamformer achieved lower SRTs than the fixed beamformer setting. Adaptive beamforming improved SRT in both CI user groups substantially by about 3 dB (bimodal group) and 8 dB (bilateral group) depending on masker type.

**Conclusions:**

CI users showed SRM that was comparable to normal hearing subjects. In listening situations of everyday life with spatial separation of source and masker, directional microphones significantly improved speech perception with individual improvements of up to 15 dB SNR. Users of bilateral speech processors with both directional microphones obtained the highest benefit.

## Introduction

Speech perception in everyday life situations is a key challenge in the treatment of hearing impairment. Regularly, background noise is present which compromises speech perception even in normal hearing listeners. Depending on the environment, acoustical properties of noise differ in terms of spectral shape and temporal envelope. Additionally, noise masker position may vary during a conversation. Binaural hearing enables localization of sound sources and perceptual separation of speech sources from irrelevant noise [1]. Spatial separation of speech source and noise source leads to better speech perception in normal hearing subjects [2]. This effect is often referred to as “spatial release from masking” (SRM). SRM is induced by head shadowing of the noise source and the binaural effects of summation and squelch. Speech perception in noise is often assessed by the measurement of speech reception threshold (SRT) at which 50% of the presented words are understood correctly. Usually, SRT measured in a condition where speech S and noise N are co-located at 0° (S_0_N_0_) is used as a reference for the calculation of SRM.

Recipients of cochlear implants (CIs) in both ears or with bimodal fitting (CI with a hearing aid in the contralateral ear) can benefit from bilateral hearing to improve speech perception in interfering noise. They also show a benefit for different masker and target positions [3–6]. Besides spatial parameters, the temporal characteristics of noise interferer affect speech perception. However, SRM in bimodal listeners is substantially poorer compared to normal hearing subjects. SRM in CI users is mainly generated by head shadowing, whereas binaural interaction effects such as the squelch effect or binaural summation provide only marginal benefit, as reported in different studies with bilateral CI users [7–9]. The limited delivery of binaural cues by current CI stimulation strategies is thought to be responsible for SRM deficits. Furthermore, the behind the ear position of the microphone combined with a spectral resolution restricted to 22 or less frequency channels prevents the use of pinna effects, which are important for front-back localization and spatial source separation [10].

**Fig 1:**
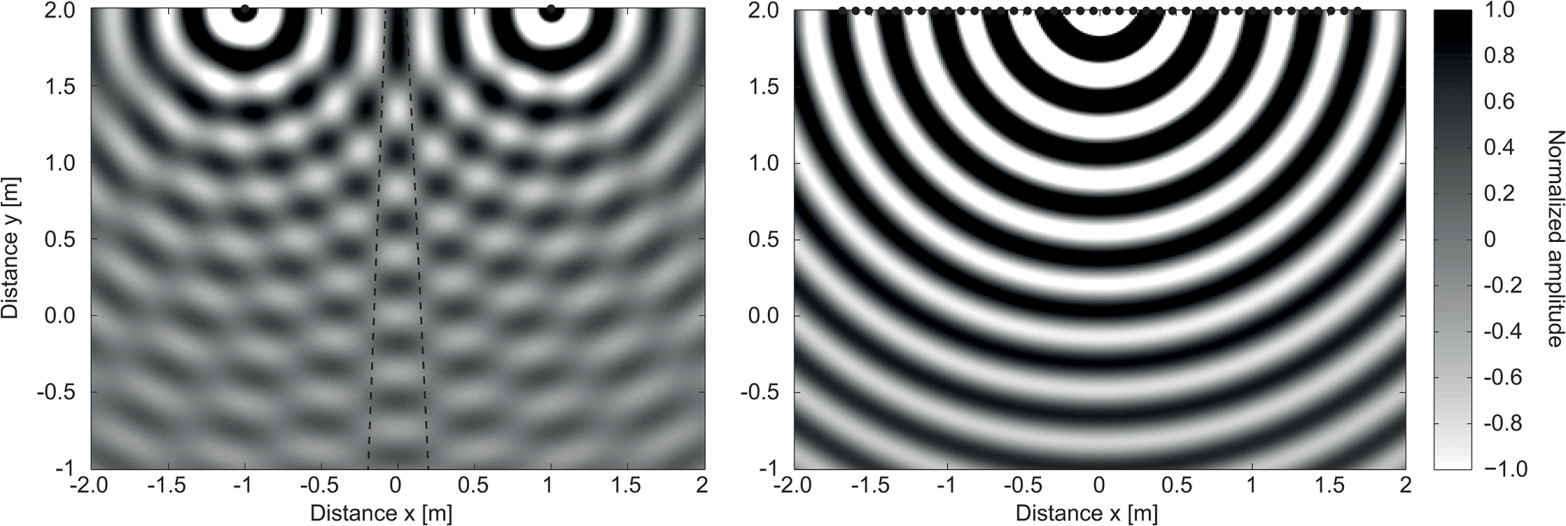
Comparison of stereophonic and wave field synthesis playback. Left: Calculated sound field generated by two sound sources (f = 1 kHz) at positions (x = ±1 m, y = 2 m) comparable to stereophonic playback (equal amplitudes). Right: Simulated wave field synthesis (n = 40 loudspeakers, Δx = 8.6 cm) corresponding to the setup installed in the present study. The result of each playback method is a virtual sound source at position (x = 0 m, y = 2 m). For stereophonic playback, the area of exact sound field is limited to a very narrow “sweet spot” (between dashed lines), whereas wave field synthesis can cover a much larger listening area.

Over the last decades, improved stimulation strategies [11,12] and new CI signal preprocessing schemes derived from hearing aid technology have increased speech perception in many listening situations. The beneficial impact of noise reduction algorithms and directional microphones achieved by combination of multiple microphones (so-called beamforming, e.g. [13]) in CI listeners was shown in different studies [14–19]. However, these investigations were conducted in static conditions with fixed positions of speech and noise during testing. Enhanced beamforming algorithms introduced in cochlear implant speech processors employ adaptive processing in order to dynamically adjust the angle of highest noise suppression to the position of a moving noise source [20]. To estimate the impact of adaptive beamforming techniques, speech perception should be assessed in situations with varying masker position during speech presentation. Conventional playback systems equipped with several fixed loudspeakers are unable to represent a moving noise source correctly. Thus, speech perception in dynamic listening situations with moving noise sources was assessed rarely so far. Using multiple noise sources or switching between discrete loudspeakers has previously been used, but since the noise source locations were discrete, the sound movement was not presented in a realistic manner [18,19,21–24]. Panning of the noise source amplitude to create virtual sound sources has also been used [25]. However, the sound field elicited by amplitude panning highly differs from the sound field of a real moving sound source, and thus differs from reality. Precise sound reproduction in terms of spatial amplitude distribution is crucial to evaluate adaptive beamforming algorithms properly. Spatial aliasing in stereophonic amplitude panning, even at low frequencies, could potentially induce amplitude errors and thus diminish the performance of the beamformer. Creating stable lateral phantom sources with amplitude panning is not always possible [26]. An example for sound field differences between amplitude panning and real source behavior is shown in Fig 1. The results of a computer simulation of a centered static source sound field (according to [27]) generated by a 1 kHz pure tone elicited by stereophonic playback is displayed in Fig 1 (left). The area with exact sound field reproduction and thus correctly perceived direction of sound incidence is limited to a very small spot (so-called “sweet spot”). Fig 1 (right) displays the calculated results of a sound field for the same signal, but generated by means of simulated wave field synthesis (WFS, [28]). The WFS driving functions were adjusted to generate the same sound source position as by stereophonic playback. It is visible that the simulated WFS sound field is equal to a single sound source covering a very large area.

As demonstrated in Fig 1, WFS represents a crucial advantage for the generation of realistic sound fields. Furthermore, controlled movement of sound sources is possible in an accurate way. We implemented a moving masker paradigm to generate a demanding listening situation resembling everyday life. In addition, two different modulation characteristics of the interfering masker were employed to imitate speech or environmental non-speech stimuli.

In summary, the aims of the present study were (1) to investigate SRM in a moving masker setup with different temporal envelopes of masker in normal hearing subjects and CI listeners, and (2) to evaluate the impact of adaptive beamforming compared to fixed directional microphones on speech perception in moving noise.

## Material and Methods

### Participants

**Fig 2:**
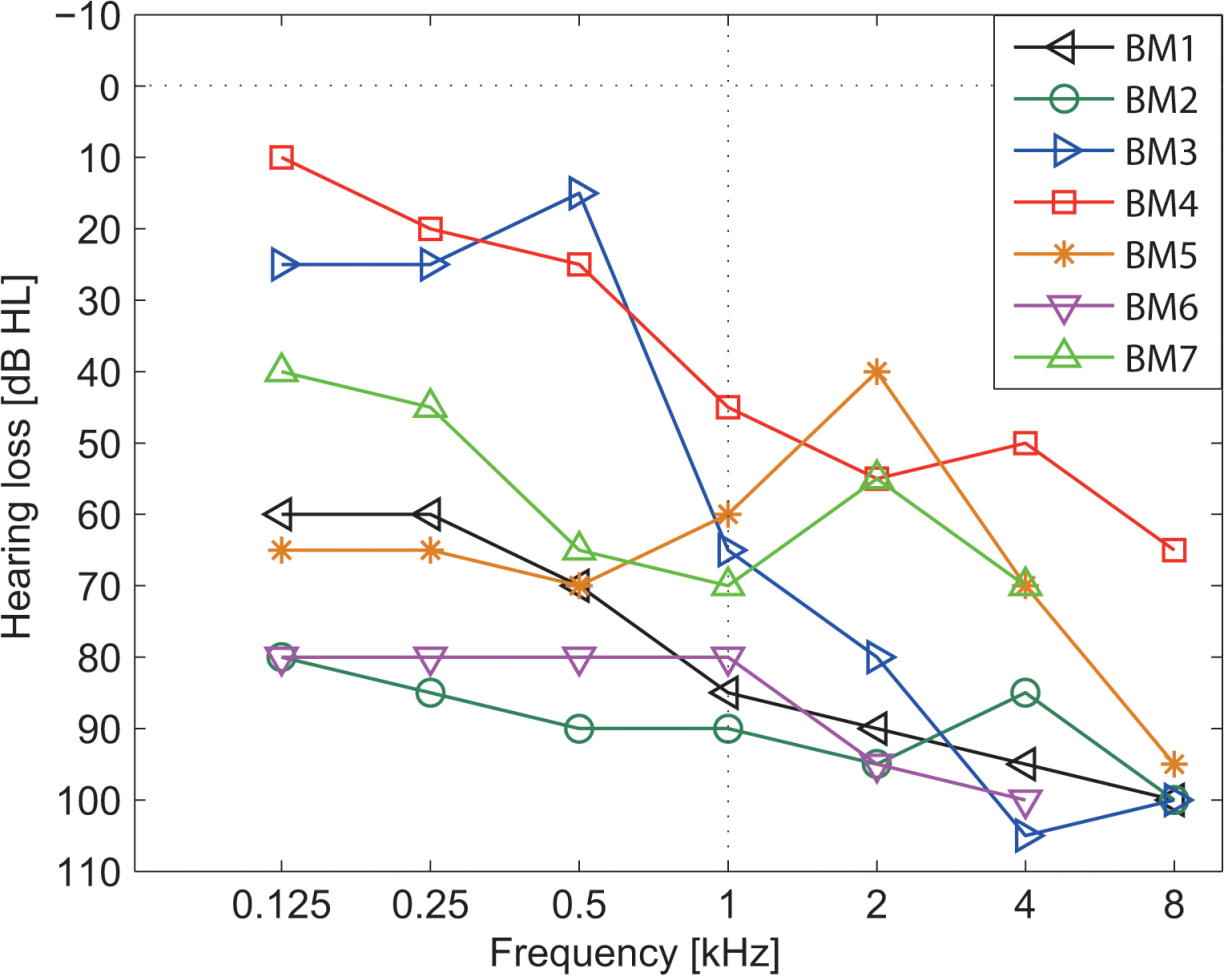
Individual pure-tone thresholds (air conduction) of the bimodal CI group (n = 7) measured in the non-implanted ear.

12 normal hearing participants (9 female, mean age 26.4 ± 5.4 years) and 14 CI users (3 female) took part in the measurements. In the CI group, 7 of the participants were bilateral CI users (mean age 49.9 ± 14.3 years) and 7 were bimodal CI users (mean age 49.6 ± 19.1 years) with a hearing aid in the contralateral ear. All CI patients were users of Cochlear CP810 speech processors and were tested in best aided condition. Demographical data of CI users is shown in Table 1. Only subjects with postlingual deafness took part in the study. Etiologies were congenital (n = 2), otitis media acuta (n = 1), ototoxic drugs (n = 1), or unknown (n = 10). In the bimodal group, hearing aid fitting was assessed by means of aided free field audiometry, whereby speech perception scores of numbers and monosyllables recognition was measured at a sound level of 65 dB SPL with the Freiburg Speech Test [29]. Individual pure-tone thresholds of the non-implanted ear derived in the bimodal group are shown in Fig 2. All subjects except for subject BM6 used a behind-the-ear hearing aid. Four subjects used Phonak hearing aids with Digital audio Zoom or VoiceZoom, one subject a Siemens Signia with directional sensitivity. Microphone settings of the hearing aids of subjects BM6 and BM7 are unknown. However, all tests with bimodal subjects were carried out with their regularly used program. In the evaluation of the benefit of the beamformer, each subject served as his own control.

**Table 1:** Study participants.

ID	Condition	Age [yrs]	Left ear	Right ear	Experience CI left [yrs]	Experience CI right [yrs]	Monosyllable score left	Monosyllable score right
BM1	bimodal	38	CI, CI512	HA, Phonak Naida	1.0	-	85%	20%
BM2	bimodal	50	HA, Siemens Signia	CI, CI24RE	-	1.1	20%	95%
BM3	bimodal	17	CI, CI512	HA, Phonak Naida V UP	2.1	-	95%	90%
BM4	bimodal	65	CI, CI24RE	HA, Phonak Versata M	1.1	-	90%	90%
BM5	bimodal	54	CI, CI422	HA, Phonak Naida V UP	0.6	-	95%	40%
BM6	bimodal	47	CI, CI512	HA, ITE (n/a)	2.8	-	65%	0%
BM7	bimodal	77	CI, CI24RE	HA, Coselgi Cmb	0.6	-	90%	15%
BL1	bilateral	48	CI, CI512	CI, CI512	1.8	1.8	85%	75%
BL2	bilateral	66	CI, CI512	CI, Ci422	1.5	0.3	80%	80%
BL3	bilateral	65	CI, CI24RE	CI, CI24RE	0.4	1.0	90%	90%
BL4	bilateral	54	CI, CI512	CI, CI24RE	1.8	1.0	95%	80%
BL5	bilateral	31	CI, CI512	CI, CI512	3.1	1.3	55%	45%
BL6	bilateral	55	CI, CI512	CI, CI24RE	2.5	0.9	80%	85%
BL7	bilateral	31	CI, CI24RE	CI24(CS)	1.6	11.3	95%	80%

### Ethics Statement

The study was approved by the local institutional review board (University of Frankfurt/Main, reference number 394/12). Subjects gave their written consent of participation and received financial compensation for their participation.

### CI Speech Processor Settings

The individual clinical standard MAP with ACE strategy [12] was used for every subject of the CI group. No additionally available processing algorithms except for the automatic gain control were active. The CP810 speech processor is equipped with two omnidirectional microphones. The speech processor offers three different settings of directional sensitivity:

*Standard*: A moderately directional microphone, with about 5 dB attenuation at 180°. This moderate amount of directionality mimics the natural ear.

*Fixed (ZOOM)*: Narrower beam towards the front than *standard* with about 5 dB attenuation at 180° compared to 0° with maximal attenuation of about 17 dB at 120°. Polar plots of *standard* and *fixed* directionality of the CP810 speech processor are shown in [30].

*Adaptive (BEAM)*: Maximum sensitivity at 0° with additional adaptive minimum sensitivity steered by beamforming towards the direction of the most intense noise. This present beamforming algorithm is a modification of the method introduced by Wouters and Vanden Berghe [17], which was designed for a two-microphone combination consisting of a directional and an omnidirectional microphone.

Speech perception measurements of the present study were conducted in the three aforementioned settings of the speech processor. The order of test conditions was randomized.

### Sound Playback Setup

**Fig 3:**
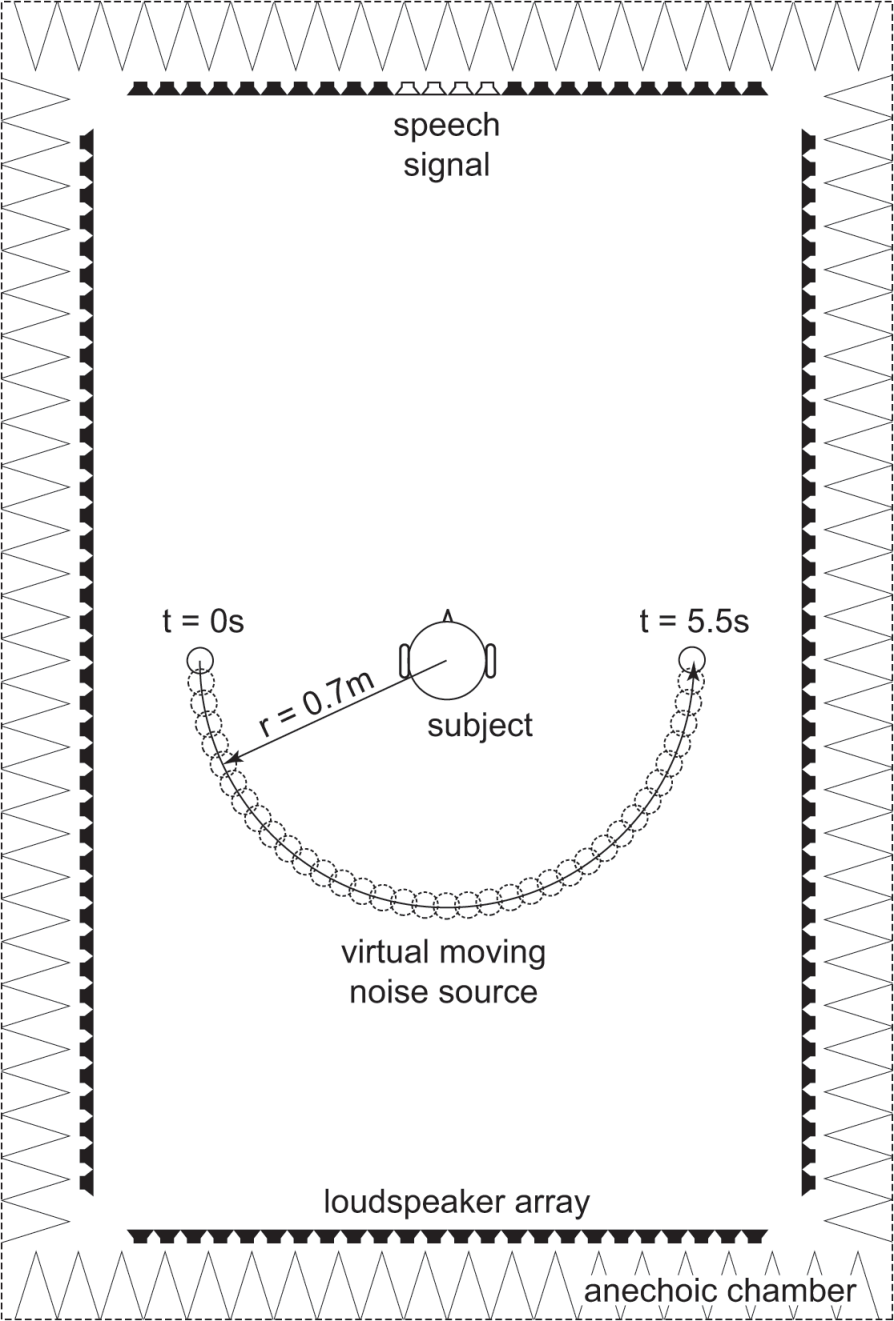
128 loudspeakers in a horizontal rectangular array used for speech in noise evaluation. Speech was always presented from 0°. Four adjacent loudspeakers were combined in parallel for speech playback to avoid distortions. The moving noise source N_move_ was generated by means of wave field synthesis.

A playback system comprising 128 custom-built loudspeakers was realized in the anechoic chamber (dimensions 4.10 x 2.60 x 2.10 m^3^, length x width x height) of our department. Loudspeakers were mounted in the horizontal plane at a height of 1.20 m. In order to comprise the frequency range of CI or hearing aid signal processing, each loudspeaker was equalized up to 10 kHz with finite impulse response (FIR) filter. Above 10 kHz, frequency response of the correction filter was set to 1. Distance between adjacent loudspeakers was 8.6 cm to achieve sufficient spatial resolution. The position of the loudspeakers inside the room is shown in Fig 3. Audio amplifiers were class D amplifier modules (Hypex UcD180HG) with passive heatsinks. A voltage divider was applied to decrease output power and reduce internal noise generated by the amplifiers. Noise floor inside the anechoic chamber with all amplifiers switched on was 33.9 dB(A).

The application of WFS [28,31] allowed for creation of virtual sound sources at almost any arbitrary position in azimuth, outside or inside of the listening room. The theory of WFS is based on a principle postulated by Huygens [32] in the 17^th^ century, which indicates that every element on a wave front produced by a sound source can be considered as the center of a new sound source. Thus, the sound field of a virtual primary source can be created by the superposition of multiple physically existent secondary sound sources (loudspeakers) with infinitesimal spacing. Virtual sound sources are realized by appropriate time delay and level adjustment of the loudspeakers.

Audio playback was realized with a standard personal computer running Ubuntu Linux version 12.04 LTS equipped with 3 RME HDSPe MADI PCI express cards with a total of 192 simultaneous playback channels. SoundScape Renderer 0.3.1 [33] was used for real-time rendering of all WFS sources. The personal computer was located in a control room to minimize noise floor in the anechoic chamber. The audio stream of 128 channels were transmitted to the anechoic chamber via MADI optical fibers.

### Stimuli

Two noise conditions, which differed in temporal envelope and spectral characteristic, were tested: (1) a continuous broadband noise with long-term spectral shape derived from an overlay of several sentences of the Oldenburg matrix test (“Olnoise”, [34]). The continuous noise was chosen to simulate environmental stimuli with low temporal modulation like vacuum cleaners or fans. (2) Test noise of the “Commité Consultatif Internationale Télégrafique et Téléfonique” (CCITT-noise) with a speech-like power density spectrum, which was amplitude modulated at a randomized modulation frequency (“Fastl-noise”, [35]). The spectral distribution of the modulating signal peaks at 4 Hz, which correlates with mean amount of spoken syllables/s of Western speech. In the following, “Olnoise” is termed “continuous noise” and “Fastl-noise” is termed “modulated noise”.

The speech reception threshold (SRT) in background noise was assessed in a customized version of the matrix test (Oldenburg sentence test, referenced as OLSA in the following) whereby the level of the noise signal was fixed (65 dB SPL) and speech level was set adaptively according to the number of words perceived correctly. The OLSA was conducted in “closed set” mode. Therefore, after presentation of each sentence the subject had to indicate on a touch screen which elements of the sentences were understood. Speech levels automatically increased when two or fewer words were perceived correctly and decreased when more than two words were correct. The step sizes for the adaptive procedure decreased with the number of inflection points as suggested by Brand and Kollmeier [36]. Each test list consisted of 20 sentences, which contained a noun, verb, numeral, adjective, and object. Each one of these words was randomly selected out of a list of 10 possible options. Because of this method of sentence construction, some sentences made no sense. This resulted in low memorability and predictability [37]. The results of the OLSA test are expressed as a specific speech reception threshold. The signal-to-noise ratio (SNR) was calculated from individuals’ SRT levels.

Compared to the “open set” OLSA procedure, the process of training subjects to get them accustomed to the voice of the talker and the test course is accelerated with the closed set method. Results obtained with the closed set method show slightly better performance than with the open set procedures, but do not show increased effects of memorability. This is because the conciseness of the OLSA sentences is very low preventing a general learning effect [38]. Speech perception was assessed for two spatial setups:

1. S_0_N_0_

Speech signal S and noise N were presented from front (0°) with a distance of 1.75 m to the listening position. Four adjacent loudspeakers of the WFS setup were used in parallel to obtain sufficient sound pressure level with negligible distortion at the listening position. In total, the width of these four speakers was 34.4 cm, which is comparable to the width of a single conventional loudspeaker used for audiometry. The increase in low frequency caused by the superposition of the four loudspeakers was compensated. The participant was placed in the far field of the loudspeakers. The duration of speech signal varied in each sentence and was about 3 seconds. Noise started 5 seconds prior to speech onset and stopped two seconds after speech offset. Overall duration was about 10 seconds.

2. S_0_N_move_

Speech signal stimuli S were presented from 0° as described in setup S_0_N_0_. The masker source was rendered with WFS [33]. Initial position of the masker was at either +90° or -90° in a distance of 0.7 m from the middle of the subjects’ head. For the bimodal group, the side of the CI (which was the left ear except for one bimodal user) was set as initial side of the masker. For the bilateral group, the side of the ear with better monosyllable score was set as initial side of the masker. For each subject, the starting direction of the noise source was kept constant in all trials.

Noise masker was presented for 5 seconds at fixed position of either ±90°. Then the masker started a smooth motion in the shape of a semi-circle from ipsilateral ear via the back to the contralateral ear with a radius of *r* = 0.7 m and a velocity *v* = 0.4 m/s (see Fig 3). This meant that the noise traversed a semi-circle of 180° in 5.5 seconds. Speech onset was 1 second after the start of masker movement. Thus, speech was present during masker movement approximately between 120° and 240°.

After one training list in S0N0 continuous noise condition, presentation of spatial configuration and noise type was randomized. Sentence lists were also randomized.

Stimuli were calibrated in free field at listening position using a sound pressure level meter NTI Audio XL1. All sounds were calibrated to same *L*_eq_ of 65 dB SPL averaged over 30 seconds. Audio files of speech stimuli were normalized to same energy (root-mean-square) as continuous noise. Therefore, continuous noise was also used for calibration of speech stimuli from 0°. Calibration was rechecked after study completion.

## Results

### Normal Hearing Group and CI Users with Directivity Setting *standard*

**Fig 4:**
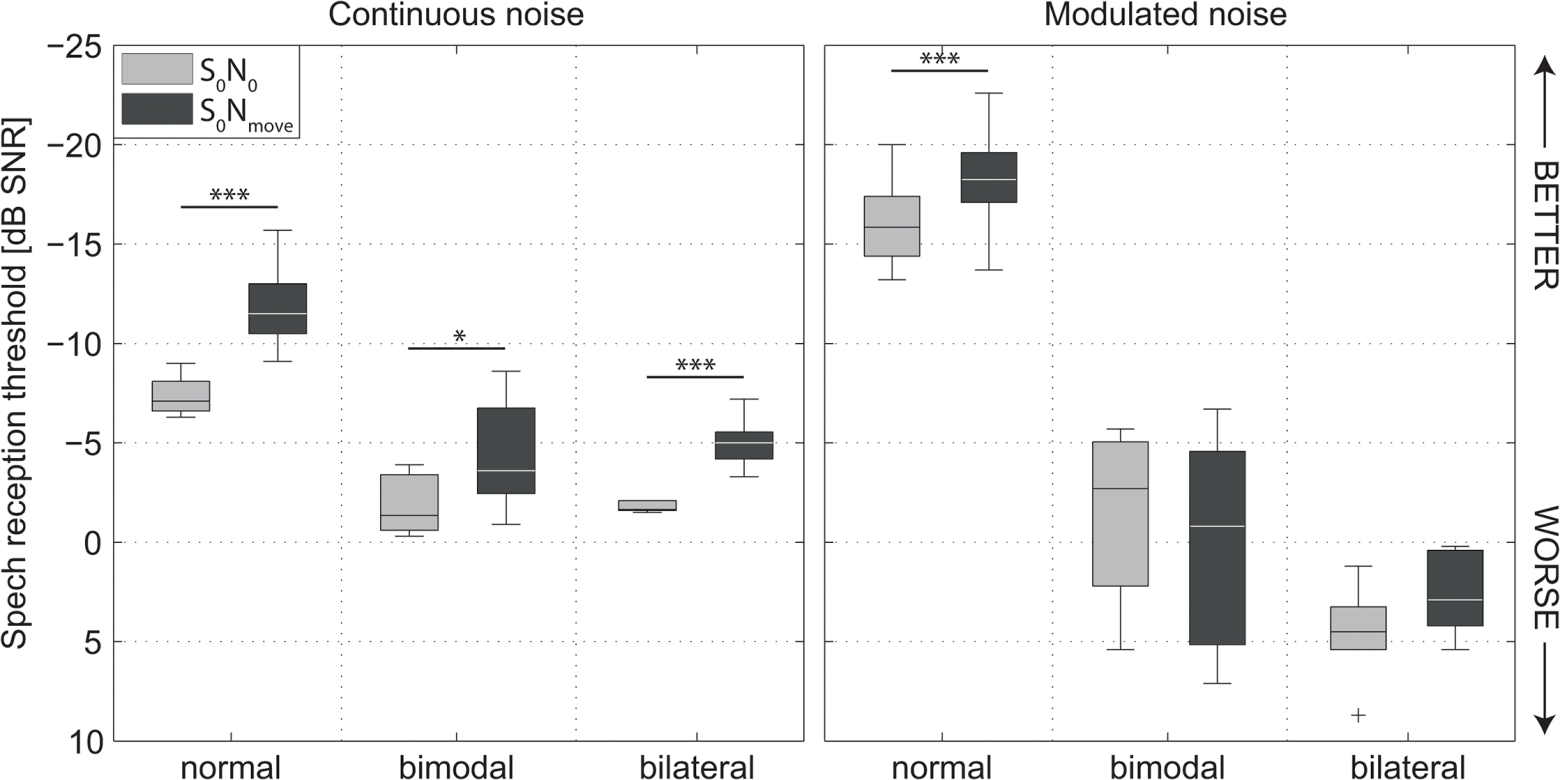
Speech reception thresholds (SRTs) of all participants in all test conditions. CI users were tested with *standard* directional setting. The asterisks indicate a statistically significant difference in SRT between S_0_N_0_ and S_0_N_move_ (*p < 0.05, **p < 0.01, ***p < 0.001).

Median, interquartile, and range values of SRT measurements of the normal hearing group and CI users with directivity setting *standard* in S_0_N_0_ and S_0_N_move_ setups are shown in Fig 4. As the *y* axis is inverted, higher performance (lower SRT) is depicted by “higher” boxplots. Outliers (defined as data points more than 1.5 box-lengths away from the median) are indicated by crosses. Individual results of the normal hearing group are shown in the S1 Table.

#### Effect of Masker Position

A *t* test for paired comparisons revealed significantly lower SRTs in S_0_N_move_ compared to S_0_N_0_ for the normal hearing control group (4.3 dB improvement, *t* = 9.98, df = 13, *p* < 0.001), the bimodal group (2.6 dB improvement, *t* = 2.86, df = 6, *p* = 0.029), and for the bilateral group (2.9 dB improvement, *t* = 9.62, df = 6, *p* < 0.001) in continuous noise. In the modulated masker condition, only the normal hearing group showed a significantly decreased SRT (2.3 dB improvement, *t* = 3.63, df = 13, *p* < 0.003) in S_0_N_move_. All subject groups benefited from a spatial separation of speech and masker in continuous noise, whereas spatial separation of modulated noise was only beneficial for normal hearing listeners.

#### Effect of Subject Group

Group differences were analyzed by analysis of variances (one-way ANOVA). In S_0_N_0_ setup, a group effect was found for both noise conditions (continuous: *F*[2,25] = 95.51, *p* < 0.001, modulated: *F*[2,22] = 157.93, *p* < 0.001). A Tukey post hoc test indicated a significant difference between normal hearing group and both CI groups for all noise conditions (*p* < 0.001). SRT in modulated noise in the bimodal group were significantly lower than in the bilateral group (*p* = 0.004).

In S_0_N_move_ setup, there was a group effect for both noise conditions (continuous: *F*[2,25] = 41.65, *p* < 0.001, modulated: *F*[2,25] = 117.19, *p* < 0.001). A Tukey post hoc test indicated a significant difference between normal hearing group and both CI groups (*p* < 0.001) in continuous and modulated noise. A tendency of lower SRT in modulated noise in the bimodal group compared to the bilateral group could not be statistically confirmed due to the high individual differences (interquartile range of about 10 dB) in the bimodal group.

Subjects with bilateral electric hearing performed worse compared to groups with acoustic or combined hearing in modulated noise condition.

#### Effect of Masker Type

SRT of the normal hearing group decreased significantly by 8.7 dB (S_0_N_0_, *t* = 22.62, df = 13, *p* < 0.001) and 6.7 dB (S_0_N_move_, *t* = 14.73, df = 13, *p* < 0.001) in modulated noise compared to continuous noise condition. In the bimodal group no effect of masker type was found in S_0_N_0_ setup whereas in S_0_N_move_ there was a significant increment in SRT of 4.6 dB in the condition with modulated masker (*t* = -3.34, df = 6, *p* = 0.016). The bilateral group showed an increased SRT in modulated noise for both masker positions (S_0_N_0_: 6.7 dB, *t* = -9.34, df = 6, *p* < 0.001; S_0_N_move_: 7.5 dB, *t* = -8.29, df = 6, *p* < 0.001).

Compared to continuous noise, amplitude modulated noise improved SRTs in normal hearing dramatically, whereas strong performance decrements were observed in CI users.

#### Spatial Release From Masking

Spatial release from masking was calculated as individual difference between SRT in S_0_N_0_ and S_0_N_move_ setup. The results are shown in Table 2. All groups showed a beneficial effect resulting from spatial masker separation. There was no statistical difference between subject groups in each noise condition. All subject groups showed a tendency of lower SRM in modulated noise. However, only the normal hearing group showed a significant difference in SRM of 2 dB between continuous and modulated noise conditions (*t* = 5.49, df = 13, *p* < 0.001).

**Table 2:** Mean spatial release from masking (SRM) and standard deviation (SD).

	Continuous noise		Modulated noise	
	SRM	SD	SRM	SD
**Normal**	4.3 dB	1.6 dB	2.3 dB	2.3 dB
**Bimodal**	2.8 dB	2.3 dB	0.8 dB	2.2 dB
**Bilateral**	3.0 dB	0.8 dB	2.1 dB	2.4 dB

### Comparison of SRT with Directional Setting *fixed* and *adaptive*

Individual and mean speech reception thresholds of CI users in S_0_N_move_ according to three different directional sensitivity settings *standard*, *fixed* (static beamformer “ZOOM”), and *adaptive* (adaptive beamformer “BEAM”) are given in Table 3. A multi-way ANOVA with factors subject group, directional sensitivity setting and noise type indicated significant main effects of directional sensitivity setting (*F*[2,70] = 14.21, *p* < 0.001) and noise type (*F*[2,70] = 50.32, *p* < 0.001) with an interaction of subject group and noise condition (*F*[1,70] = 4.43, *p* = 0.039). A Tukey post hoc analysis confirmed lower SRTs in setting adaptive compared to *fixed* (*p* = 0.015) and *standard* (*p* < 0.001) setting. Post hoc analysis showed significantly lower SRTs in the bilateral group in continuous noise for *adaptive* compared to *fixed* (5.1 dB improvement, *p* = 0.001) and to *standard* (7.8 dB improvement, *p* < 0.001) settings. SRT in the *adaptive* setting was found to be significantly lower than the *standard* setting in modulated noise (6.6 dB improvement, *p* = 0.007). No differences between directional sensitivity settings were found in the bimodal group, probably generated by large between subject variability.

**Table 3:** Individual results of bimodal and bilateral subjects in S_0_N_move_ condition for directional sensitivity settings *standard*, *fixed*, and *adaptive*.

Subject	Continuous noise [dB SNR]			Modulated noise [dB SNR]		
	standard	fixed	adaptive	standard	fixed	adaptive
BM1	-6.3	-8.9	-13.1	-4.6	-5.6	-11.1
BM2	-0.9	-1.7	-3.4	7.1	5.2	0.4
BM3	-8.6	-7.9	-10.3	-6.7	-7.8	-7.8
BM4	-3.6	-4.6	-4.7	-4.5	-6.2	-4.1
BM5	-2.4	-3.8	-8.4	5.0	0.7	-1.1
BM6	-2.6	-3.9	-4.3	5.2	2.9	2.8
BM7	-6.9	n/a	-8.2	-0.8	n/a	-2.1
**MEAN**	**-4.5**	**-5.1**	**-7.5**	**0.1**	**-1.8**	**-3.2**
**SD [dB]**	**2.8**	**2.7**	**3.5**	**5.6**	**5.4**	**4.8**
BL1	-5.0	-7.4	-12.3	0.3	-1.1	-0.4
BL2	-4.0	-7.0	-11.0	5.4	0.7	-2.7
BL3	-3.3	-5.4	-11.8	2.9	0.8	0.6
BL4	-4.8	-6.3	-8.5	0.7	2.7	-2.7
BL5	-7.2	-6.4	-16.1	3.6	-1.0	-11.5
BL6	-5.4	-9.4	-14.9	4.4	-4.2	-6.3
BL7	-5.6	-11.7	-15.0	0.2	-8.8	-5.9
**MEAN**	**-5.0**	**-7.7**	**-12.8**	**2.5**	**-1.6**	**-4.1**
**SD [dB]**	**1.3**	**2.2**	**2.7**	**2.1**	**3.8**	**4.1**

#### Individual Differences

**Fig 5:**
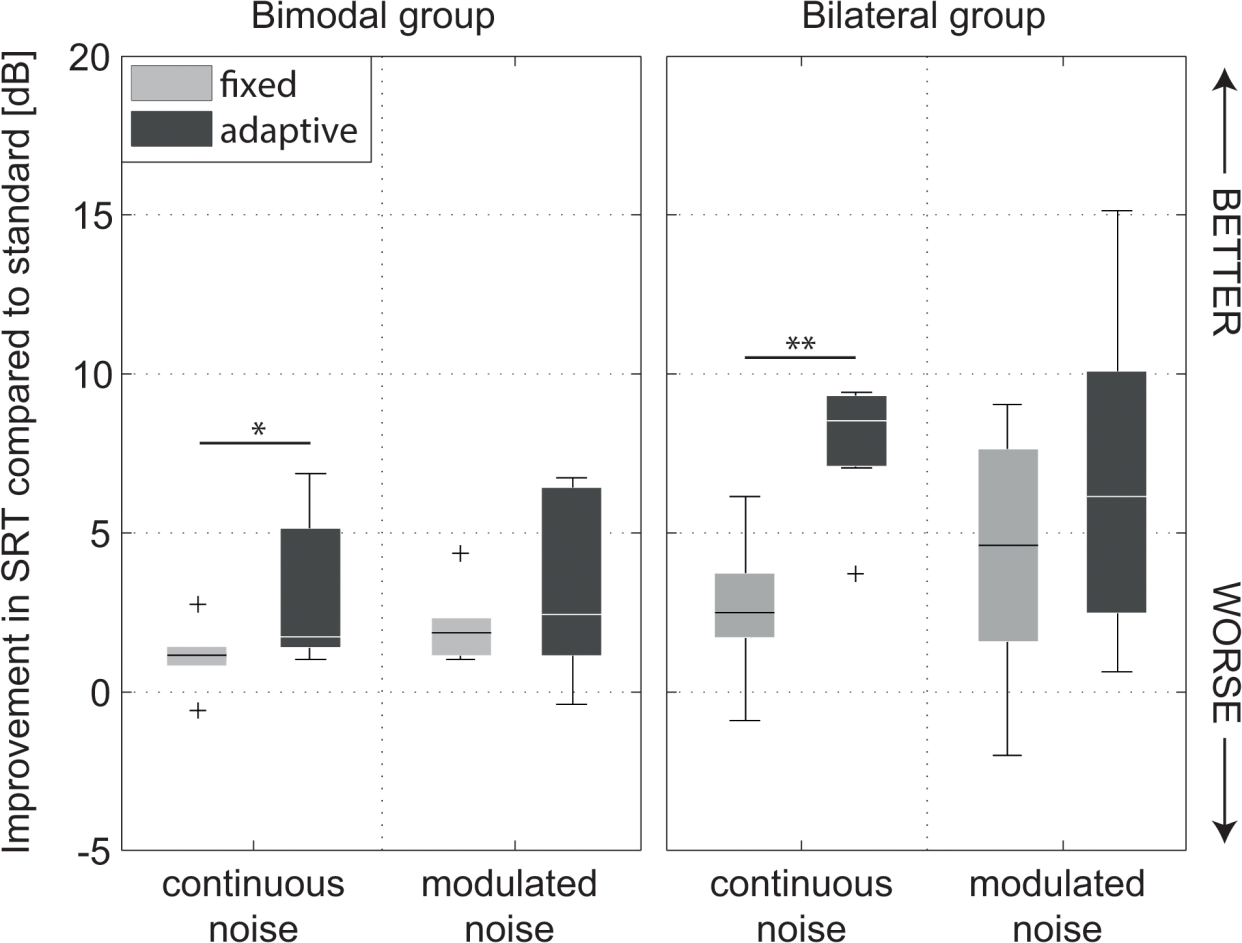
Improvement of SRT in beamforming conditions *fixed* and *adaptive* in S_0_N_move_. *Standard* directionality condition was used as reference. Left: bimodal CI recipients, right: bilateral CI recipients (COCHLEAR CP810). The asterisks indicate a statistically significant difference in SRT between setting *fixed* and *adaptive* (*p < 0.05, **p < 0.01).

Individual subjects SRTs were found to be highly independent of directional sensitivity settings (Table 3). This is expected to be due to a number of factors including performance of the non-implanted ear in bimodal users, duration of deafness, duration of CI experience and auditory nerve survival. In order to determine the individual impact of different directional sensitivity settings, the difference in SRT between the *standard* and *fixed* or *adaptive* settings was calculated. The results are illustrated as SRT improvement in Fig 5. Individual benefit ranged from -2 dB to 9 dB in *fixed* setting and -0.4 dB to 15.1 dB in the *adaptive* setting compared to standard.

Significantly higher improvement of SRT was observed in the *adaptive* compared to the *fixed* setting in the bimodal group (2.2 dB, *t* = -2.87, df = 5, *p* = 0.035) and in the bilateral group (5.1 dB, *t* = -5.53, df = 6, *p* = 0.001) in continuous noise. A comparison between bilateral and bimodal group in the *adaptive* setting revealed significantly higher improvement in the bilateral group (*t* = -4.072, df = 12, *p* = 0.002) whereas for *fixed* setting only a tendency of higher improvement was observed.

In modulated noise, significant differences between different directional sensitivity settings and subject groups were absent.

## Discussion

Spatial release from masking (SRM) in cochlear implant patients was investigated in previous studies solely in fixed loudspeaker setups (an overview of different studies is given in Culling et al. [39]) with measurements predominantly in S_0_N_+90_ or S_0_N_-90_ setups. The present study examined speech reception thresholds and SRM in a continuously moving noise source setup with cochlear implant users.

The first part of the discussion in the following compares SRM in moving noise setup to results from studies with a fixed masker position. Furthermore, the effect of the two different masker types and the contribution of binaural cues in cochlear implant users are discussed.

The second part of discussion describes the potential impact of adaptive beamforming on speech perception in cochlear implant users.

### Control Group and CI Users (*standard* Setting)

#### Acoustic vs. Electric Listening

In listeners with normal hearing, the presence of short temporal gaps in the envelope of masking noise leads to strong SRT improvement. Compared to continuous masking, the beneficial effect amounts to about 7 dB with modulated masking noise [35]. The explanation for this ameliorative effect is the ability of normal hearing listeners to pick up signal information inside masking noise gaps. During these temporal gaps, signal-to-noise ratio is higher compared with continuous masking. This effect has been termed the “glimpsing effect” [40].

The beneficial effect of a co-located fluctuating masker in normal hearing subjects was reaffirmed in the present study group (8.7 dB). The glimpsing effect was 2 dB lower with moving masker (6.7 dB) compared to the static masker condition.

Most hearing-impaired patients suffer from deteriorated gap listening abilities manifested in higher thresholds, which presumably do originate from slow rates of recovery [41]. The absence of the glimpsing effect in bilateral CI users and subjects using electric-acoustic stimulation was also recently shown by Rader et al. [6]. Likewise, in the present study, speech reception thresholds in bilateral CI users in modulated noise were higher compared to continuous noise condition in both S_0_N_0_ and S_0_N_move_ setups. The reason for the absence of the glimpsing effect in bilateral CI users is still unclear.

Concerning the bimodal subject group, limited aided hearing in the contralateral ear may enhance individual performance due to a residual glimpsing effect. Compared with the bilateral CI subject group, bimodal listeners showed a tendency to benefit from acoustic hearing in the contralateral ear (1 dB, S_0_N_0_ setup). The large variability in SRT in modulated masker condition observed in the bimodal group is mainly explained by the large variations seen in the individual pure tone audiograms (Fig 2). Depending on the amount of useful residual hearing, the success of hearing aid fitting differs, which is reflected by the large variation of aided monosyllable scores ranging in between 0% and 90% (Table 1). The influence of the amount of residual hearing is conveyed by a significant negative correlation of aided monosyllable score (hearing aid only) and SRT in best aided bimodal condition found in the S_0_N_move_ condition with modulated masker (*r* = -0.77, *p* < 0.05). Since this correlation was absent in continuous masker condition, potentially a residual glimpsing effect is responsible for this effect observed in the bimodal listener group.

#### Comparison with Results of SRM with Fixed Masker Position

The standard paradigm for measuring SRM is a comparison of S_0_N_0_ with fixed masker S_0_N_90_ or S_0_N_180_ (e.g. [42]). In the following, studies which measured SRM with fixed masker paradigm, are compared with a moving masker paradigm to discuss differences between both approaches.

Plomp and Mimpen [42] reported SRM of 9.8 dB for S_0_N_90_ and 1.4 dB for S_0_N_180_ in normal hearing subjects applying a continuous masker of a spectral shape resembling the speech signal. Vom Hoevel and Platte [43] reported similar results for S_0_N_90_ but a higher SRM of 4 dB in S_0_N_180_. In the present study, mean SRM in the normal hearing group was 4.3 dB for continuous noise. This result is in line with Plomp and Mimpen and vom Hoevel and Platte since an SRM lower than in S_0_N_90_ but higher than in S_0_N_180_ setup would be expected in the moving masker setup.

Carhart et al. [44] measured SRM as a function of modulation characteristics of the masker. In this experiment, one of the tested masking noises was set to a modulation frequency of *f*_m_ = 4 Hz and a modulation of *m* = 62%. This setting is comparable to the modulated masker used in the present study. As outlined in the results section of the present study, the SRM difference between continuous and modulated noise was 2 dB. This result is in line with Carhart et al. who identified a difference of 1.7 dB between white noise and modulated white noise (S_0_N_90_ speaker setup). The reduced SRM in modulated noise compared to a continuous masker could be explained by the “glimpsing effect” [40]. Since the ability to listen to short temporal gaps leads to decreased SRT in the S_0_N_0_ setup of about 8 dB, the effect of source separation on speech perception is lower compared to continuous noise conditions.

Spatial release from masking in CI users was investigated in several previous studies with fixed masker positions. In the following, studies with maskers at the sides of or behind the listener are compared with the results of the present study.

Schleich et al. [8] reported average SRM of 3 dB (mean of S_0_N_0_ vs. S_0_N_-90_ and S_0_N_+90_) in a group of bilateral CI patients (TEMPO+ speech processor, MED-EL) in continuous noise. Van Hoesel and Tyler [7] reported a mean SRM of 4.5 dB found in a population of bilateral CI users with either Cochlear Sprint or Esprit speech processors, which utilized a fixed moderately directional microphone. The same holds for the CP810 speech processor in setting *standard* (everyday preprocessing condition), which was used in the present study. Therefore, SRM in S_0_N_90_ is expected to be about 1.5 dB higher in this population of CI users with a moderately directional microphone compared to speech processors with omnidirectional microphone.

#### Head Shadowing and Binaural Cues

According to previous studies concerned with SRM in fixed masker position, lower SRTs compared with co-located masker measurements were expected due to head shadow and squelch effect in the moving masker paradigm. In the present study, the impact of head shadow on interaural level differences was not constant over the presentation of the moving masker.

At a masker position of 180°, head shadowing is absent. At this position, interaural time differences and interaural level differences of speech and masker are expected to be close to zero and only summation effects potentially improve speech perception. However, in the present study, the actual duration of presentation of the masker at a location of about 180° was below 100 ms. Therefore, substantial summation effects were unlikely in such a short time period.

### Comparison of SRT with *fixed* and *adaptive* Directional Sensitivity in CI Users

The *adaptive* beamforming algorithm substantially decreased SRTs in both patient groups compared to the *standard* microphone directionality by 3.0 dB (bimodal group) and 7.8 dB (bilateral group) in the continuous noise condition. Even in the modulated noise condition, a large effect was present (3.3 dB bimodal and 6.6 dB bilateral CI users). In everyday listening situations, the occurrence of modulated noise is more frequently expected. Therefore, it can be expected that the current implementation of the beamforming algorithm will provide additional benefit compared to *standard* directional sensitivity in everyday life challenging listening situations.

#### Effect of Patient Group

Comparing *standard* and *adaptive* beamforming conditions, mean individual improvement in the bilateral group is about twice as high as in the bimodal group for both continuous noise (3.0 vs. 7.8 dB) and modulated noise (3.3 vs. 6.6 dB). The binaural combination of adaptive beamforming seems to double SRT improvement. It is obvious that bimodal patients receive only half of the beneficial effect, since in the moving noise source setup the support of beamforming is only present for half of the speech stimulus sentence. In contrast, wearing bilateral adaptive beamformers ensured noise reduction during the whole sentence presentation when the noise source travels around the back of the CI listener.

#### Effect of Directional Sensitivity Setting

The three different microphone sensitivity settings applied a different amount of directional sensitivity to the front. The difference between *standard* setting (“everyday”) and *fixed* (“ZOOM”) is the angle of minimal sensitivity at 120°, and the level of attenuation at this direction. The *adaptive* (“BEAM”) setting applied different frontal directionality compared to *fixed* setting. Therefore, it remains difficult to distinguish whether the amount of SRT improvement was due to the ability of “BEAM” to adapt to the noise source or by generally enhanced frontal directionality. However, the results of the present study clearly showed the benefit of microphone directionality for speech perception in free field conditions.

One limitation in today’s CI speech processors and most hearing aids is the independent operation of each device. It is expected that bilateral CI users will benefit from synchronization of both CI speech processors. With bilaterally synchronized processors in S_0_N_move_ setup, the *adaptive* setting on the side opposite to the noise could employ the microphone input signal to form a better reference signal. Kokkinakis and Loizou [45] already reported an improved speech perception of 20% in S_0_N_90_ setup with two synchronized BEAM algorithms compared to independently running speech processors. Buechner et al. [46] reported about 2 dB lower SRTs in unilateral CI users with a binaural 3^rd^ order beamformer (two synchronized Phonak Ambra hearing aids connected to the CI via auxiliary input) in a quasi-diffuse noise field S_0_N_±70, ±135,180_.

#### Comparison of the Present Results with Previous Studies

The performance of adaptive beamforming algorithms was previously reported by Wouters and Vanden Berghe [17], and Spriet et al. [18]. In both studies, SRT was measured in small groups of experienced unilateral CI users in fixed S_0_N_90_ setup. Differing from the present study where two omnidirectional microphones were applied, beamforming was implemented using the combination of directional and omnidirectional microphone.

Wouters and Vanden Berghe reported a mean improvement in SRT (lists of numbers) of 9.5 dB in speech-weighted noise and 10 dB for ICRA noise compared to the standard directional microphone. These results were obtained by feeding the preprocessed stimuli recorded via BTE processor positioned at the artificial ear of a dummy head into the auxiliary input of a clinical speech processor.

Spriet et al. investigated the *adaptive* setting “BEAM” implemented in the Cochlear Freedom speech processor (predecessor of the CP810 device). SRT improvement measured in 5 unilateral CI users for the Dutch/Flemish LIST sentences [47] was reported as high as 13.4 dB (speech-weighted noise) and 15.9 dB (multi-talker babble noise).

Mean improvement in the aforementioned studies with adaptive beamforming was higher than in both CI subject groups of the present study. This difference may be explained by the position of the noise source, which was fixed at 90° in those studies. Furthermore, patients were tested monaurally and the noise source was located at the implanted ear. In contrast to monaural test conditions in the aforementioned studies, all subjects in the present study were binaural listeners (CI+HA or CI+CI). Binaurally aided subjects can benefit from head shadowing during the moving noise masker presentation (except for 180°), independent from the setting of directional sensitivity. Thus, there is less room for improvement from using beamforming algorithms when comparing binaural and unilateral listening.

## Conclusions

Spatial release from masking (SRM) was present in hearing-impaired listeners using cochlear implants either bimodally or bilaterally. A semi-circular motion of a masker in the back during speech presentation from zero degrees azimuth decreased SRM compared to a fixed masker position at 90°. The amount of benefit obtained from SRM (about 3 to 4.5 dB) was comparable between bimodal and bilateral CI users as well as normal hearing subjects.

The results of the present study demonstrated the impact of advanced signal processing applied in CI processor devices in listening situations with spatial separation of source and noise masker. Compared to fixed microphone sensitivity, adaptive beamforming yielded improvements of up to 15 dB SNR especially in moving masker setups. Bilaterally worn CI devices employing adaptive beamforming obtained the largest improvement. However, beamforming algorithms are known to work best in anechoic conditions. Therefore, further investigations of everyday listening situations in reverberant rooms are of interest. A combination of beamforming with other preprocessing (e.g. noise reduction) could potentially improve speech perception, especially in reverberant conditions.

As demonstrated by Rader et al. [6], temporally modulated noise remains the most challenging listening condition for CI recipients. Even with beamforming, the difference between normal hearing subjects and bilateral CI users was as high as 20 dB SNR.

In the future, the implementation of binaural signal processing strategies with synchronized adaptive beamforming could further improve speech perception in complex noise environments.

## Supporting Information

S1 Table Individual results of normal hearing subjects with mean and standard deviation (SD). (XLS)
